# Correction to: Diversity and evolution of surface polysaccharide synthesis loci in Enterobacteriales

**DOI:** 10.1038/s41396-021-01181-9

**Published:** 2022-01-19

**Authors:** Kathryn E. Holt, Florent Lassalle, Kelly L. Wyres, Ryan Wick, Rafał J. Mostowy

**Affiliations:** 1 Department of Infectious Diseases, Central Clinical School, Monash University, Melbourne, VIC Australia; 2 London School of Hygiene and Tropical Medicine, London, UK; 3 Department of Infectious Disease Epidemiology, School of Public Health, Imperial College London, London, UK; 4 Malopolska Centre of Biotechnology, Jagiellonian University, Krakow, Poland

**Keywords:** Molecular evolution, Genomics, Microbial genetics

Correction to: *The ISME Journal* 14:1713–1730 10.1038/s41396-020-0628-0, published online 6 April 2020

In this article was given Cronobacter sakazakii incorrectly as Citrobacter sakazakii.

The original article has been corrected.

